# Comparison of phenomics and cfDNA in a large breast screening population: the Breast Screening and Monitoring Study (BSMS)

**DOI:** 10.1038/s41388-023-02591-z

**Published:** 2023-01-24

**Authors:** Justin Stebbing, Panteleimon G. Takis, Caroline J. Sands, Lynn Maslen, Matthew R. Lewis, Kelly Gleason, Karen Page, David Guttery, Daniel Fernandez-Garcia, Lindsay Primrose, Jacqueline A. Shaw

**Affiliations:** 1 Department of Surgery and Cancer, Imperial College London, Du Cane Road, Hammersmith, London, W12 0NN UK; 2 School of Life Sciences, Faculty of Science and Engineering, ARU, East Road, Cambridge, CB1 1PT UK; 3 National Phenome Centre and Imperial Clinical Phenotyping Centre & Section of Bioanalytical Chemistry, Division of Systems Medicine, Department of Metabolism, Digestion and Reproduction, IRDB Building, Imperial College London, Hammersmith Campus, London, W12 0NN UK; 4 Leicester Cancer Research Centre, Department of Genetics and Genome Biology, University of Leicester, Robert Kilpatrick Clinical Sciences Building, Leicester Royal Infirmary, Leicester, LE2 7LX UK

**Keywords:** Breast cancer, Diagnostic markers

## Abstract

To assess their roles in breast cancer diagnostics, we aimed to compare plasma cell-free DNA (cfDNA) levels with the circulating metabolome in a large breast screening cohort of women recalled for mammography, including healthy women and women with mammographically detected breast diseases, ductal carcinoma in situ and invasive breast cancer: the Breast Screening and Monitoring Study (BSMS). In 999 women, plasma was analyzed by nuclear magnetic resonance (NMR) and Ultra-Performance Liquid Chromatography-Mass Spectrometry (UPLC-MS) and then processed to isolate and quantify total cfDNA. NMR and UPLC-MS results were compared with data for 186 healthy women derived from the AIRWAVE cohort. Results showed no significant differences between groups for all metabolites, whereas invasive cancers had significantly higher plasma cfDNA levels than all other groups. When stratified the supervised OPLS-DA analysis and total cfDNA concentration showed high discrimination accuracy between invasive cancers and the disease/medication-free subjects. Furthermore, comparison of OPLS-DA data for invasive breast cancers with the AIRWAVE cohort showed similar discrimination between breast cancers and healthy controls. This is the first report of agreement between metabolomics and plasma cfDNA levels for discriminating breast cancer from healthy subjects in a true screening population. It also emphasizes the importance of sample standardization. Follow on studies will involve analysis of candidate features in a larger validation series as well as comparing results with serial plasma samples taken at the next routine screening mammography appointment. The findings here help establish the role of plasma analysis in the diagnosis of breast cancer in a large real-world cohort.

## Introduction

Breast cancer (BC) is the most frequent cause of death among women after lung cancer, worldwide [1]. Current diagnosis is largely based on a physical examination, mammographic and other imaging and histopathological assessment of tissue biopsy, complemented by blood tests for the detection of specific antigens and/or proteins [2, 3]. Early diagnosis significantly increases long-term survival rates [4]. However, more sensitive and breast cancer-specific biomarkers are required for early detection of aggressive disease.

Use of cfDNA was first described over 60 years ago [5]. Elevated levels are seen in cancer in part due to reduced DNase activity [6–8]. Elevated levels of cfDNA in plasma have been suggested for the diagnosis of breast cancers and qualitative tests have demonstrated increased cfDNA integrity/size [9–11]. However elevated levels of cfDNA are also sometimes observed in benign breast disease [12], reducing its specificity for cancer. Certain patterns in cfDNA (e.g. mutations, loss of heterozygosity (LOH), hypermethylation) have the potential to provide specific markers and have also been investigated [13–15]. We have previously described that that patient-specific circulating tumor (ctDNA) analysis can detect early evidence of progression up to 2 years ahead of imaging [16].

Altered metabolism is one of the key hallmarks of cancer. The development of sensitive, reproducible and robust bioanalytical tools such as NMR and mass spectrometry (MS) techniques has allowed us to explore its role [17, 18] in conjunction with other new methods. We have previously shown that metabonomics identifies excess energy expenditure pathways perturbed during chemotherapy for breast cancer [19] and have suggested new therapeutic approaches that focus on metabolism [20]. Either individually or grouped as a metabolomic profile, detection of metabolites can be carried out in the same plasma samples as cfDNA analysis. We have thus explored the potential of using both cfDNA and the metabolome together, in a large cohort of women recalled for mammography at Imperial College Healthcare NHS Trust, including healthy women and women with early mammographically detected breast cancer. We also compared results to a second independent series of healthy controls from the AIRWAVE study. Together the use of cfDNA and metabolomics, when used as a translational research tool, can provide a link between the laboratory and clinic.

## Results

The demographics and clinical metadata of the 1185 individuals analyzed in this study are reported in the Supplementary Table 1 comprising 999 from the BSMS study and 186 female individuals recruited from AIRWAVE (AW II).

### NMR spectroscopy

**Fig. 1:**
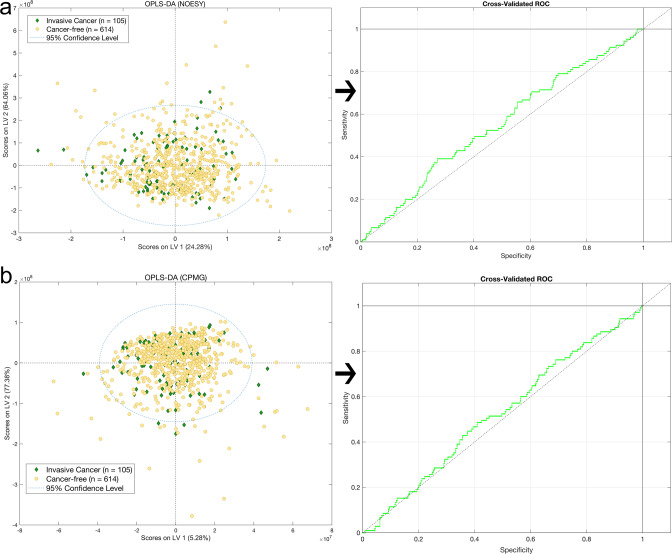
OPLS-DA analysis of plasma ^1^H-NMR global profiling data between Invasive breast cancer vs. cancer-free subjects. Scores plots and the ROC curves of the OPLS-DA analyses between cancer-free vs. Invasive breast cancer subjects from **a** NOESY and **b** CPMG NMR spectral data.

In the BSMS cohort OPLS-DA of plasma ^1^H-NMR global profiling data (1D-NOESY and CPMG) between patients diagnosed with invasive breast cancer and cancer-free subjects, did not show significant discrimination (Table 1, Fig. 1a, b). Similar non-significant discrimination was found between groups for the comparison between benign vs. in situ, invasive cancer vs. benign, invasive cancer vs. in situ and cancer-free vs. all breast cancer groups. Similar results, with poor discrimination accuracy (<60%, Table 1) between all studied groups (Supplementary Fig. 2) were obtained for OPLS-DA modeling of the plasma NMR targeted data (19 metabolites and 112 lipoproteins).

**Table 1:** Summary of OPLS-DA models comparing Invasive BC cases versus all other groups based upon untargeted/targeted NMR and MS assays with their corresponding cross-validated (CV) accuracy and AUC values.

Models	Accuracy (CV) (%)			AUC (CV)		
**NMR**	**NOESY**	**CPMG**	**Targeted (metabolites/lipoproteins)**	**NOESY**	**CPMG**	**Targeted (metabolites/lipoproteins)**
Invasive BC vs Cancer-free	53.4	50.8	50.1/38.8	0.55	0.53	0.47/0.36
Invasive BC vs in situ	55.3	52.5	53.1/63.0	0.31	0.44	0.51/0.41
Invasive BC vs Benign	50.3	50.7	48.3/40.8	0.49	0.54	0.44/0.38
Invasive BC vs Healthy (AW)	–	–	89.1/86.3	–	–	0.94/0.93
In *situ* vs Healthy (AW)	–	–	90.2/74.3	–	–	0.95/0.78
Benign vs Healthy (AW)	–	–	88.2/75.7	–	–	0.95/0.79
**LC–MS**	**HILIC**+ **(HPOS)**^**a**^	**Lipid RPC**+ **(LPOS)**	**Lipid RPC− (LNEG)**	**HILIC**+ **(HPOS)**^**a**^	**Lipid RPC**+ **(LPOS)**	**Lipid RPC− (LNEG)**
Invasive BC vs Cancer-free	67.1	64.8	64.2	0.62	0.58	0.58
Invasive BC vs In situ	62.5	62.7	60.6	0.58	0.59	0.55
Invasive BC vs Benign	56.4	61.6	57.3	0.54	0.59	0.54
Invasive BC vs Disease/Medication-free	69.5	67.8	66.4	0.66	0.60	0.61
Disease/ Medication-free (subgroup 1) vs Disease/Medication-free (subgroup 2)	85.8	75.3	70.6	0.90	0.74	0.74
Invasive BC vs Disease/Medication-free (subgroup 1)	75.5	70.0	72.3	0.77	0.69	0.71
Invasive BC vs Disease/Medication-free (subgroup 2)	41.6	39.8	40.4	0.33	0.30	0.31

Model groupings comprise; Invasive BC: subjects diagnosed with invasive BC (*n* = 105); Cancer-free: subjects without invasive BC, in situ and benign diseases (*n* = 614); In situ: subjects with in situ cancer (*n* = 40); Benign group: subjects with benign breast disease (*n* = 214); Disease/Medication-free: subjects without BC or any other disease and being under no medication (*n* = 288); Diseases/ Medication-free (subgroup 1): subset of Disease/Medication-free group, discriminated from invasive BC with high accuracy by MS assays models (*n* = 237); Diseases/ Medication-free (subgroup 2): subset of Disease/Medication-free group, but predicted as invasive BC group with high accuracy by MS assays models (*n* = 51); Healthy (AW): healthy female subjects from an independent cohort from the AIRWAVE study (*n* = 186).

^a^HILIC+ results of the fitted models are after the removal of lidocaine features.

Taking advantage of NMR data reproducibility between spectrometers and spectra collection centers [21], we also compared invasive cancer patients with data generated as part of the AIRWAVE study, comprising an independent cohort of female healthy individuals (*n* = 186). In particular, the targeted datasets from both studies (i.e. the absolute concentration values of 19 metabolites and 112 plasma lipoproteins) were employed and used to build the corresponding MVA models. Initially, unsupervised Principal Component Analysis (PCA) was performed on diseases-free and healthy AIRWAVE individuals’ datasets from both studies to test the feasibility of coupling the two independent datasets. PCA score plot (Supplementary Fig. 3a) from the 19 metabolites concentrations showed a perfect classification between healthy AIRWAVE versus BSMS diseases-free individuals. Further examination of loadings plots (Supplementary Fig. 3b) revealed that glucose and lactic acid concentrations were significantly different between the 2 study cohorts, where glucose and lactic acid values were higher and lower, respectively, in BSMS diseases-free individuals (Supplementary Fig. 3c, d). This could be attributed to the sample collection time points, nutritional habits and/or physical exercise between individuals from each cohort, amongst possible factors. Nevertheless, glucose and lactic acid were removed from both datasets, and the new PCA results indicated an overlap without any significant classification trends between BSMS and AIRWAVE samples, allowing us to employ them for further supervised MVA analyses. It should be noted that the lipoproteins datasets were highly overlapped for both studies (Supplementary Fig. 3e) and they were employed for further analyses as such.

The supervised OPLS-DA analysis of the 17 metabolites dataset (excluding glucose and lactic acid) for BSMS patients with invasive breast cancer versus the AIRWAVE healthy subjects showed high classification accuracy (Table 1) of the two groups (Supplementary Fig. 4a, b) and one-way ANOVA calculated *p*-values after Benjamini-Hochberg correction [22] indicated citric acid, acetic acid, leucine, histidine, glycine, glutamine, pyruvic acid and creatinine as discriminative biomarkers (Supplementary Fig. 4c). The same analysis for the 112 plasma lipoproteins provided a good classification of invasive cancer patients versus healthy AW subjects (Table 1, Supplementary Fig. 5) and 17 lipoprotein classes appeared to significantly change (*p* < 0.05) between the 2 classes (Supplementary Table 2). Following the same strategy, OPLS-DA models were constructed for the comparison between benign vs healthy (AIRWAVE) (Supplementary Fig. 6a) and in situ vs healthy (AW) (Supplementary Fig. 6b) and their performance is summarized in Table 1. Results indicated again high classification accuracies for the benign vs. healthy (AW) and in situ vs healthy (AW) models based upon the 17 metabolites concentration datasets. The produced loadings from the models suggested several metabolites as potential biomarkers, such as pyruvic acid, citric acid, leucine, histidine, glycine, glutamine and creatinine (Supplementary Fig. 6c, d). It is noteworthy that although the mean age of BSMS breast cancer and AW subjects were significantly different (Supplementary Table 1), Pearson correlation analysis of all plasma metabolites concentrations with subjects’ age indicated an insignificant contribution of age to the measured values (Supplementary Fig. 7) in the present datasets.

### UPLC–MS

**Fig. 2:**
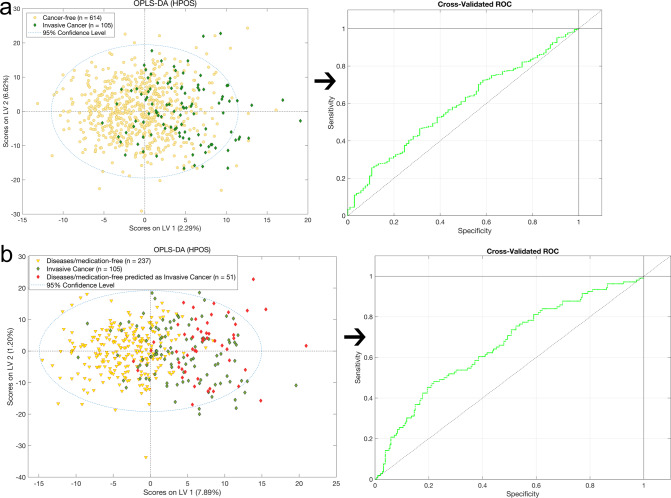
OPLS-DA analysis of MS HILIC+ (HPOS) data between cancer-free vs. invasive breast cancer subjects and their resulting subgroups. **a**. Scores plot and ROC curve of the OPLS-DA analysis between Cancer-free vs. Invasive breast cancer subjects from the MS HILIC+ (HPOS) assay data. **b**. Scores plot and the ROC curve of the OPLS-DA analysis [MS HILIC+ (HPOS) assay] between Invasive breast cancer vs. Diseases/medication-free subjects (*n* = 288), where the two observed subgroups are colored differently; those predicted as Invasive Cancer are depicted as red diamonds and the rest of the Diseases/medication-free subjects are depicted as inverted yellow triangles.

Similarly, OPLS-DA showed no significant discrimination between any sample class pairings for all LC–MS assays. In particular, the statistical models based upon the lipidomic profile of plasma samples for both positive and negative ionization modes, exhibited similar discrimination accuracy between invasive cancer and cancer-free subjects (accuracy = 64%), whereas the models from the benign vs. in situ, invasive cancer vs. benign, invasive cancer vs. in situ and cancer-free vs. the rest of the types of breast cancer groups showed lower discrimination accuracy values (i.e. <60%) (Table 1, Supplementary Fig. 8). However, a moderate discrimination accuracy (AUC = 0.65 and accuracy = 76.5%) was observed between the invasive cancer and the cancer-free control group from the HILIC+ dataset. An examination of the extracted loadings data from the supervised OPLS-DA analysis showed that the most weighted HILIC+ features leading to the observed discrimination, corresponded to lidocaine, most likely explained by contamination of several plasma samples by local anesthetic during the blood sampling procedure. When we removed HILIC+ lidocaine features and repeated the MVA analysis the model showed less accuracy in discriminating the two groups (AUC = 0.62 and accuracy = 67.0%) in agreement with the lipidomic profile (Table 1 and Fig. 2a).

**Fig. 3:**
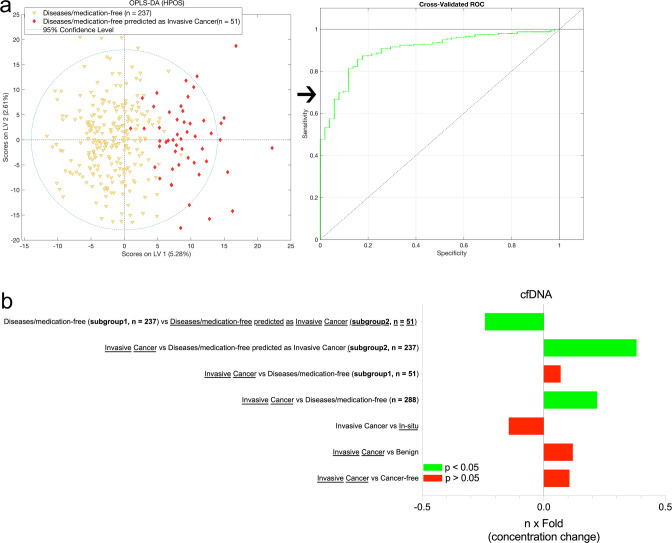
OPLS-DA analysis of MS HILIC+ (HPOS) data between diseases/medication-free subjects subgroups and univariate statistics of cfDNA data. **a** Scores plot and the ROC curve of the OPLS-DA analysis [MS HILIC+ (HPOS) assay] between Diseases/medication-free subjects subgroup 1 (*n* = 237) vs. Diseases/medication-free subjects subgroup 2 (*n* = 51) consisted of those predicted as Invasive Cancer. **b** The cfDNA *n* x Fold concentration changes between the studied groups. The *n* × Fold was calculated by the equation: $n \times {\rm{Fold}} = {\rm{log}}_2\left( {\frac{{{\rm{median}}\;{\rm{of}}\;{\rm{group}}\;1}}{{{\rm{median}}\;{\rm{of}}\;{\rm{group}}\;2}}} \right)$. Moreover, one-way ANOVA analysis coupled with t-test was performed for the determination of the statistically significant (*p* < 0.05) differences of the observed cfDNA concentration changes for each case. For each comparison, cfDNA concentration is higher in the underlined group.

Having considered lidocaine contamination of the samples, we further stratified the 614 cancer-free controls, comparing 288 reported as having no drugs intake and/or other disease with the other 326 subjects. Subsequently, we isolated this disease/medication-free group and we re-evaluated all MVA analyses for both UPLC-MS and NMR data. This was undertaken to avoid any confounding in the data owing to the presence of features corresponding to drug related compounds or to metabolites relating to other diseases that cancer-free subjects were experiencing during the blood sampling period. This OPLS-DA model for invasive cancer vs. disease/medication-free subjects indicated a slightly higher discrimination accuracy (+3%) for all UPLC–MS assays (Table 1 and Fig. 2b). When exploring the predicting ability of our models, 51 of the 288 plasma samples from the diseases/medication-free healthy controls, were predicted as invasive cancer with accuracy >85% based on their metabolic data (Table 1 and Fig. 3a).

However, the supervised OPLS-DA analysis of the diseases/medication-free vs. the diseases/medication-free predicted as invasive cancer samples showed high discrimination accuracy, namely, 86%, 76 and 71% for HILIC+, Lipid RPC+ and Lipid RPC- MS assays, respectively (Table 1). When this group of 51 control subject were excluded highly predictive models were produced from the diseases/medication-free (without those predicted as Invasive Cancer) vs. invasive cancer plasma samples, with accuracy values 76%, 70 and 73% for HILIC+, Lipid RPC+ and Lipid RPC− MS assays, respectively.

### Plasma cfDNA analysis

Initially, total cfDNA levels in all blood samples from BSMS were employed for multiple univariate ANOVA analyses, comparing the total cfDNA concentration between each group of subjects as for the metabolomics data (Fig. 3b). All univariate analyses of the cfDNA concentration corroborate the obtained results from the MS based MVA models. The total cfDNA concentration was significantly higher in invasive breast cancer vs. the diseases-free subjects, whereas the cases of cancer-free and benign tumors vs. invasive cancer samples showed no significant differences (Fig. 3b). In addition, there was no significant difference in concentration between patients with invasive and in situ cancer. Of note, the 51 diseases/medication-free subjects (subgroup 2), that were classified as “cancer like” by HILIC+, Lipid RPC+ and Lipid RPC− LC–MS assays respectively also had a significantly higher cfDNA concentration (*p* = 0.002) compared to the rest of the healthy controls (*n* = 237), whereas non-significant differences were observed vs. the invasive cancer samples. In addition, the subgroup of 237 diseases-free subjects (subgroup 1) had significantly lower cfDNA concentration vs. the invasive cancer (Fig. 3b). Consequently, cfDNA results were in total agreement with the LC-MS metabolomics data. It should be noted that Pearson correlation analysis (*r* = 0.068) of plasma cfDNA measured values with subjects’ age indicated insignificant contribution of age to the cfDNA differences between the studied groups.

As expected, the MVA analysis of the combined cfDNA and LC–MS datasets—since their agreement—produce superior OPLS-DA models i.e., with higher discrimination accuracy (see MVA results of HILIC+ and cfDNA combined datasets in Supplementary Fig. 9).

## Discussion

We report the metabolomic and cfDNA analysis of a large cohort of sequential plasma samples from 999 women attending for routine breast screening and validation with an independent cohort of 186 healthy women from the AIRWAVE study. Our main findings demonstrate the utility of cfDNA quantification here. This represents a real-world cohort, and results of this comprehensive work exemplify the challenges of establishing such a complex composite biomarker panel since the resulting accuracy of the signature derived from the UPLC-MS analysis was only moderate (AUCs between 0.62 and 0.76).

Several metabolomics studies have attempted to detect the breast cancer fingerprint in serum and plasma [1, 23, 24], showing high accuracy in models (AUC > 0.9), which discriminate breast cancer from healthy subjects. The majority of the models described in the aforementioned studies are derived by MS plasma or tissue analyses with a maximum of 100 advanced breast cancer and 100 controls, although another NMR-based metabolomic study employing a large serum/plasma cohort succeeded in monitoring and predicting BC relapse (accuracy = 71%) and discriminating early BC from metastatic BC patients (accuracy = 85%) [25]. Here, our large cohort analysis represents a much earlier cancer stage with greater power based on the larger sample size (999 women). NMR untargeted metabolomics data were incapable of discriminating/fingerprinting any of the patient groups (Fig. 1) in this screening population. Moreover, using a targeted approach nineteen metabolites and 112 lipoproteins concentrations extracted by NMR data, were also statistically insignificant among the studied groups (Supplementary Fig. 1). It is noteworthy that many plasma metabolites quantified herein are reported to change in invasive BC (e.g. l-glutamine, l-valine, creatine etc.) [1, 23, 24]. However, in this large cohort of early screen detected breast cancers none of these metabolites exhibited statistically significant variation in concentration (Supplementary Fig. 1). Such ‘negative data’ serves to reinforce the importance of performing screening studies in larger cohorts. Strikingly, our results are in agreement with a very recent study, where it was shown that NMR metabolomic data were multi-disease specific for patients risk stratification except from breast cancer [26]. Nevertheless, it is notable that the measured concentration of several plasma metabolites (i.e. creatine, histidine, valine, alanine and tyrosine) was found slightly (but not significantly) elevated in the plasma samples of women with invasive BC (Supplementary Fig. 1), which is in accordance to published literature [23, 27].

An advantage NMR spectroscopy is in its high reproducibility (provided that sample collection, preparation and spectra acquisition parameters are the same for all cases) [21], which can allow meaningful comparisons between datasets acquired from different cohorts. With this in mind, we constructed MVA models that discriminated invasive cancer, in situ and benign samples for an independent cohort of healthy women with high accuracy based upon the calculated absolute concentrations of 17 plasma metabolites as well as of 112 lipoproteins. Loadings of the models with high classification accuracy, provided several potential biomarkers which many of them were in line with the aforementioned literature. Namely, results showed an alteration of amino-acids and TCA circle metabolism in the invasive and in situ cancer subjects since in both cases there was a decrease of citric acid and an increase of histidine and glutamine [23, 27].

In addition, the increase of pyruvic acid in the plasma of cancer patients implies the altered glucose metabolism due to the presence of cancer cells (Warburg effect) [28, 29]. Several plasma lipoproteins were also observed to significantly change (Supplementary Table 2) consistent with evidence that breast cancer is influenced by environmental factors and lipoprotein levels in turn have a strong relationship with diet [30]. Data on the specific lipoproteins we have identified are lacking, and merits further investigation.

The employment of UPLC-MS lipidomic and small molecule metabolites profiling provided improved discrimination accuracy between invasive BC and healthy controls (UPLC–MS assays mean accuracy = 65.4% and NMR assays mean accuracy = 51%). Attempting to reduce the MS data “noise” due to any medication or other diseases of the healthy controls, we focussed on analysis of 288 disease and medication-free subjects, which provided an improved but still not high, classification accuracy from the invasive BC. However, MS data from the 288 diseases/medication-free subjects identified a subgroup of 51 that were commonly predicted as invasive BC patients from all MS assays compared to the rest (*n* = 237).

Of note, these data are in agreement with data for plasma cfDNA concentration, which we have shown previously to be associated with progression free survival, response rate and overall survival in patients with metastatic breast cancer [3, 31]. Clinical follow up revealed no unusual features for this group of 51 healthy subjects and all were confirmed as disease free at a census date of November 2019 suggesting that these features do not necessarily characterize a circulating cancer phenotype. Additionally, it has been shown recently that cfDNA is a significant biomarker of aging [32], however, in our study plasma cfDNA measured values showed insignificant contribution of age to the cfDNA differences between the studied groups.

Importantly, our screening study was carried out at a single site working to good clinical practice as quality assurance and following a validated standard operating procedure for plasma sample collection and processing helping to minimize any variation due to preanalytical processing. This is both an advantage and a limitation. Future studies will require standardization between institutions: for example, use of lidocaine as an anesthetic was found as a metabolite in our analyses and other external factors (e.g. diet) are well known to influence metabolomic findings. We also used cfDNA not circulating tumor DNA (ctDNA) due to the cost issues in a large diagnostic cohort such as this. Further studies may wish to include this after cfDNA analysis.

In aggregate, we describe here comparative analysis of plasma cfDNA with the metabolome in a large cohort of women recalled for mammography at Imperial College Healthcare NHS Trust, including healthy women and women with early detected breast cancer, ductal carcinoma in situ and invasive breast cancer. We did not find significant differences between groups for all metabolites, but found higher plasma cfDNA levels in invasive cancers than all other groups. We then stratified the supervised OPLS-DA analysis and total cfDNA concentration showing high discrimination accuracy between invasive cancers and healthy controls. We also compared OPLS-DA data for invasive breast cancers to a second independent control group of healthy individuals from the AIRWAVE study and found similar discrimination between breast cancers and healthy controls.

Our results not only confirm that standardization of collection and processing of biospecimens is central to reliable metabolomics studies but highlight the importance of control groups selection criteria for the -omics comparative studies. It is noteworthy that all univariate analyses of cfDNA concentration corroborate results from the MS based MVA models. To our knowledge, this is the first report of agreement between molecular phenomics (i.e., metabolomics) and plasma cfDNA levels for discriminating breast cancer from healthy subjects in a screening population. Follow on studies will involve analysis of candidate features in a larger validation series as well as comparing results with serial plasma samples taken at the next routine screening mammography appointment, but we provide foundations for its role in the diagnostic pathway for breast cancer.

## Materials and methods

### Patients and samples

We recruited individuals from the Breast Screening and Monitoring Study (BSMS) who were recalled from mammography. The study protocol was approved by the Riverside Research Ethics Committee (Imperial College Healthcare NHS Trust; Tissue Bank Ethics/REC reference numbers: 12/LO/2019; 13/LO/1152; R10015-16A; 07/Q0401/20) and conducted in accordance with Good Clinical Practice Guidelines and the Declaration of Helsinki. All patients gave written informed consent prior to participation and were over 18 years of age. 20 ml blood was taken into K2 EDTA tubes (BD Biosciences) and processed to recover plasma and buffy coat within 2 h of collection and stored at −80 °C for subsequent extraction of cfDNA and germline DNA as described previously [10]. The cohort included individuals with no breast disease, and women with biopsy confirmed benign breast disease, carcinoma in situ and those with invasive breast cancer. Driven by the LC-MS multivariate analyses (see below statistical methods) as well as clinical metadata (Supplementary Table 1), we formed several subgroups of samples due to the presence of features from medication (e.g., lidocaine, etc.). Furthermore, an additional subgroup was formed from the cancer/medication-free samples that was statistically classified as invasive breast cancer within high accuracy. This was also driven by the cfDNA assay results.

A second independent control group of healthy individuals was also analyzed from women recruited from the AIRWAVE study (MREC/13/NW/0588). The AIRWAVE Health Monitoring Study was established to evaluate possible health risks associated with the use of TETRA, a digital communication system used by the police forces and other emergency services. This is an ongoing long-term observational study following up the health of police officers and staff across the United Kingdom, with the ability to monitor both cancer and non-cancer health outcomes through data linkage. 53,280 participants have been recruited between June 2004 and March 2015 with a response rate averaging 50% of employees in participating forces. At baseline, participants completed an enrollment questionnaire (sent via routine administration or the occupational health service), or a comprehensive health screening performed locally, or both. Screened participants have now been followed-up for 7.5 years on average.

Each recruited individual provided a single EDTA 7 mL blood sample for subsequent plasma isolation and storage at −80 °C. This cohort was used for the validation of the cancer/medication-free group, aiming at testing its NMR-based model robustness/predictive accuracy, and as an external (independent) cancer/medication-free cohort versus invasive cancer samples for the detection of any biomarkers.

### Ultra-performance liquid chromatography-mass spectrometry (UPLC-MS) − ^1^H Nuclear Magnetic Resonance (NMR) spectroscopy

Plasma samples for UPLC-MS and NMR analyses were prepared and data acquired as published previously [33–35]. For UPLC-MS, the separation of lipophilic analytes by reversed-phase chromatography (lipid RPC) and the separation of hydrophilic analytes (e.g., polar and charged metabolites) by hydrophilic interaction liquid chromatography (HILIC) took place. MS positive and negative electrospray ionization modes produced lipid positive and negative (lipid RPC+ and lipid RPC− respectively) and HILIC positive (HILIC+) datasets. Solution ^1^H-NMR spectra of all samples were acquired using a Bruker IVDr 600 MHz spectrometer (Bruker BioSpin) operating at 14.1. Further details about the quality control of both UPLC-MS and NMR data, metabolites quantification as well as experimental procedures can be found in supplementary materials.

### Extraction and quantitation of plasma cfDNA

Cell-free DNA was isolated from 4 ml of blood plasma with the MagMAX Cell-free DNA Isolation Kit (Thermo Fisher Scientific) on the Kingfisher Flex instrument (Thermo Fisher Scientific) using the MagMAX cfDNA-4mL-Flex.bdz protocol and processed according to the manufacturer’s instructions.

### Statistical analyses – multivariate/univariate statistics

Multivariate statistical (MVA) models, specifically Orthogonal Partial Least Squares–Discriminant Analysis (OPLS-DA) of NMR and UPLC-MS metabolomics data and clinical metadata were generated between study participants with invasive cancer (*n* = 105), in situ (*n* = 40) and benign breast disease (*n* = 214), and imaging or biopsy confirmed cancer-free controls (*n* = 614). Modeling was performed in MATLAB (MathWorks, version R2019b), using the PLS_Toolbox version 8.7.1 (2019) (Eigenvector Research, Inc., Manson, WA, USA 98831; software available at http://www.eigenvector.com). All multivariate statistical models and their metrics were produced after cross-validation. Any correlation of metabolomics/cfDNA data with subjects’ age/height/weight (see Supplementary Table 1) was performed by refitting each multivariate model after adding each variable into the model and calculating its accuracy.

For all studied groups, age/height/weight were not appeared as statistically significant variables. Variables loadings data (i.e., metabolites’ LC–MS/NMR features) and Variable Importance in Projection (VIP) scores from each multivariate OPLS-DA model were used to initially evaluate any significant feature (i.e., any metabolite that could drive the classification between studied groups). VIP scores estimate the importance of each variable in the projection used in a PLS model and is often used for variable selection. A variable with a VIP Score close to or greater than 1 (one) can be considered important in given model. Variables with VIP scores significantly less than 1 (one) are less important and might be good candidates for exclusion from the model [36]. Nevertheless, each variable’s statistical significance (i.e. metabolites and lipoproteins concentration) was further tested by univariate (ANOVA) analyses via built in MATLAB functions (https://uk.mathworks.com/help/stats/one-way-anova.html). Any reported *p*-values were corrected for false discovery rate (FDR) (applying Benjamini-Hochberg FDR correction [22] using “fdr_bh” function (https://www.mathworks.com/matlabcentral/fileexchange/27418-fdr_bh).

## Supplementary information

SUPPLEMENTAL MATERIAL

## Data Availability

The datasets generated and/or analyzed during the current study are not publicly available due to individuals’ privacy reasons but are available from PGT and JAS on reasonable request and formal legal agreement.

## Code availability

Any software/code employed in the study is available to download from the links reported in the Methods section: “Statistical analyses – Multivariate/Univariate statistics”.
