# Supplementary material for: Comparison of phenomics and cfDNA in a large breast screening population: the Breast Screening and Monitoring Study (BSMS)
Source: Oncogene. 2023 Jan 24;42(11):825–32. doi: 10.1038/s41388-023-02591-z (PMC10005936; doi:10.1038/s41388-023-02591-z)
Supplement: Supplementary file 1 — SUPPLEMENTAL MATERIAL [file 41388_2023_2591_MOESM1_ESM.pdf]

Comparison of phenomics and cfDNA in a Breast Screening Population

Justin Stebbing[1,2†], Panteleimon G. Takis[3†*], Caroline J. Sands[3], Lynn Maslen[3], Matthew R. Lewis[3], Kelly Gleeson[1], Karen Page[4], David Guttery[4], Daniel Fernandez-Garcia[4], Lindsay Primrose[4], and Jacqueline A Shaw[1,4.]

[†]Joint first authors

1. Department of Surgery and Cancer, Imperial College London, Du Cane Road, Hammersmith, London, W12 0NN, United Kingdom.
2. School of Life Sciences, Faculty of Science and Engineering, ARU, East Road, Cambridge, CB1 1PT, United Kingdom.
3. National Phenome Centre and Imperial Clinical Phenotyping Centre & Section of Bioanalytical Chemistry, Division of Systems Medicine, Department of Metabolism, Digestion and Reproduction, IRDB Building, Imperial College London, Hammersmith Campus, London, W12 0NN, United Kingdom.
4. Leicester Cancer Research Centre, Department of Genetics and Genome Biology, University of Leicester, Robert Kilpatrick Clinical Sciences Building, Leicester Royal Infirmary, Leicester, LE2 7LX, United Kingdom.

*Address for correspondence:

Dr. Panteleimon G. Takis
email address: p.takis@imperial.ac.uk

Table of Contents

Experimental Details ***S3***

Ultra-Performance Liquid Chromatography-Mass Spectrometry (UPLC-MS) **S3**

^{1}H Nuclear Magnetic Resonance (NMR) Spectroscopy **S3**

Extraction and quantitation of plasma cfDNA **S4**

Supplementary Table 1. .... ***S5***

Supplementary Figure 1 .... ***S6***

Supplementary Figure 2 .... ***S7***

Supplementary Figure 3 .... ***S8***

Supplementary Figure 4 .... ***S9***

Supplementary Figure 5 .... ***S10***

Supplementary Table 2 .... ***S11***

Supplementary Figure 6 .... ***S12***

Supplementary Figure 7 .... ***S13***

Supplementary Figure 8 .... ***S14***

Supplementary Figure 9 .... ***S15***

Supplementary References ***S16***

Experimental Details

Ultra-Performance Liquid Chromatography-Mass Spectrometry (UPLC-MS)

Plasma samples were prepared and data acquired as published previously [1, 2]. In brief, a 50 µL aliquot was taken from each 1 mL sample and protein removed by addition of organic solvent, mixing, and centrifugation, to yield a homogenous supernatant. Prepared samples were subjected to ultra-performance liquid chromatography (ACQUITY UPLC, Waters Corp., Milford, MA, USA) for the separation of lipophilic analytes (e.g., complex and neutral lipids) by reversed-phase chromatography (lipid RPC) and the separation of hydrophilic analytes (e.g., polar and charged metabolites) by hydrophilic interaction liquid chromatography (HILIC). UPLC analyses were coupled to mass spectrometry (Xevo G2-S TOF mass spectrometers, Waters Corp., Manchester, UK) via a Z-spray electrospray ionization source operating in both positive and negative ion modes to produce lipid positive and negative (lipid RPC+ and lipid RPC- respectively) and HILIC positive (HILIC+) datasets. For quality control assessment and pre-processing, a pooled QC sample was prepared by combining equal parts of each study sample. A pooled QC sample was acquired every 10 study samples throughout the sample analysis. In addition, the pooled QC was diluted to seven different concentrations and replicates acquired at each concentration at the beginning and end of each set of sample analyses to assess response to dilution. Feature extraction was performed in XCMS [3] and in-house scripts applied for elimination of potential run-order effects and feature filtering [4]. Only features measured with high analytical quality (relative standard deviation (RSD) in pooled QC samples less than 20%, dilution series Pearson correlation to dilution factor greater than 0.8, RSD in study samples greater than 1.1* RSD in pooled QC) were retained and put forward for biological analysis [4].

^{1}H Nuclear Magnetic Resonance (NMR) Spectroscopy

The general procedure of NMR samples preparation is described in detail in Dona et al. [5]. Briefly, NMR samples were prepared into 96-well plates by adding 350 µL of plasma sample to each well of the 96-well plate and mixed with 300 µL of serum/plasma buffer. 600 µL of the mixture was

transferred into NMR tubes of the SampleJet. Solution ^1H NMR spectra of all samples were acquired using a Bruker IVDr 600 MHz spectrometer (Bruker BioSpin) operating at 14.1 T and equipped with a 5 mm PATXI H/C/N with ^2H-decoupling probe including a z-axis gradient coil, an automatic tuning-matching (ATM) and an automatic refrigerated sample changer (Sample-Jet). Temperature was regulated to 310 ± 0.1 K. For each blood NMR sample, 3 NMR experiments were acquired in automation: a general profile ^1H NMR water presaturation experiment using a one-dimensional pulse sequence where the mixing time of the 1D-NOESY experiment is used to introduce a second presaturation time, a spin echo edited experiment using the Carr-Purcell-Meiboom-Gill (CPMG) pulse sequence which filters out signals from fast T_2 relaxing protons from molecules with slow rotational correlation times such as proteins and other macromolecules, and a 2D J-resolved experiment. Free induction decays of all 1D-spectra were multiplied by an exponential function equivalent to 0.3Hz line-broadening before applying Fourier transform. All Fourier transformed spectra were automatically corrected for phase and baseline distortions, and referenced to the TSP singlet at 0ppm. For quality control assessment a pooled QC sample was similarly prepared by combining equal parts of each study sample and pooled QC samples were acquired regularly throughout the sample analysis. As for LC-MS data, NMR data quality control took place by in-house software, so as to employ spectra with the highest quality [4, 5]. In addition, quantification of 19 metabolites and 112 lipoproteins were performed by the in *vitro* diagnostics platform (IVDr) from Bruker Biospin (www.bruker.com)[6].

Extraction and quantitation of plasma cfDNA

4ml of plasma was combined with 2.5ml DNA lysis buffer and 30ul Dynabeads™ MyOne™ Silane beads and subjected to an automated procedure with a final elution step providing 100ul of cfDNA. DNA was isolated from 200µl white blood cells (germ line DNA control (gDNA)) as described previously[7]. Quantitation and quality check of total cfDNA and gDNA was performed using the Qubit™ dsDNA BR Assay kit (Thermo Fisher Scientific) and Agilent Tapestation HS D5000 (Agilent) according to manufacturer’s instructions.

Supplementary Table 1.

Patients	Mean age (yrs)	Being on contraceptives	Being on oestrogen replacement therapy	1st degree relative with BC	Mean Height (cm)*	Mean Weight (kg)*	Smoking status	Breast side (L: Left) (R: Right (LR: Both)	Lymph/ Vascular invasion	Mean Size of largest tumour (mm)	HER2 Status	DCIS/LCIS presence	Calcium presence
Invasive BC<br>(n = 106)	57.8	NO: 100<br>(94.3 %)<br>YES: 5<br>(4.7 %)<br>N/A: 1<br>(< 1 %)	NO: 98<br>(92.4 %)<br>YES: 7<br>(6.6 %)<br>N/A: 1<br>(< 1 %)	NO: 90<br>(84.9 %)<br>YES: 14<br>(13.2 %)<br>N/A: 2<br>(1.9 %)	N/A: 55<br>164	N/A: 59<br>68.9	Smoker: 11 (10.4 %)<br>Ex-smoker: 7 (6.6 %)<br>Non-smoker: 81 (76.4 %)<br>N/A: 7 (6.6 %)	L: 47<br>R: 55<br>LR: 4	NO: 27<br>YES: 6<br>N/A: 73	N/A: 70<br>19.78	POS: 13<br>NEG: 62<br>N/A: 31	YES: 50<br>NO: 29<br>N/A: 27	YES: 32<br>NO: 49<br>N/A: 25
Benign<br>(n = 214)	55.2	NO: 209<br>(97.7 %)<br>YES: 3<br>(1.4 %)<br>N/A: 2<br>(< 1 %)	NO: 202<br>(94.4 %)<br>YES: 11<br>(5.1 %)<br>N/A: 2<br>(< 1 %)	NO: 180<br>(84.1 %)<br>YES: 26<br>(12.1 %)<br>N/A: 8<br>(3.7 %)	N/A: 119<br>163	N/A: 127<br>67.5	Smoker: 28 (13.1 %)<br>Ex-smoker: 13 (6.1 %)<br>Non-smoker: 171 (79.9 %)<br>N/A: 2 (< 1 %)	L: 110<br>R: 102<br>LR: 2	-	N/A: 208<br>18.14	-	-	-
In situ<br>(n = 40)	59.1	NO: 40<br>(100 %)<br>YES: 0<br>(0 %)<br>N/A: 0<br>(0 %)	NO: 39<br>(97.5 %)<br>YES: 1<br>(2.5 %)<br>N/A: 0<br>(0 %)	NO: 30<br>(75 %)<br>YES: 9<br>(22.5 %)<br>N/A: 1<br>(2.5 %)	N/A: 20<br>165	N/A: 21<br>68.13	Smoker: 0 (0 %)<br>Ex-smoker: 3 (7.5 %)<br>Non-smoker: 34 (85 %)<br>N/A: 3 (7.5 %)	L: 20<br>R: 20<br>LR: 0	-	-	-	-	-
Cancer-free<br>(control) (n = 614)	50.4	NO: 596<br>(97.1 %)<br>YES: 11<br>(1.8 %)<br>N/A: 7<br>(1.1 %)	NO: 569<br>(92.7 %)<br>YES: 35<br>(5.7 %)<br>N/A: 10<br>(1.6 %)	NO: 523<br>(85.2 %)<br>YES: 71<br>(11.6 %)<br>N/A: 20<br>(3.2 %)	N/A: 331<br>163	N/A: 343<br>67.57	Smoker: 94 (15.3 %)<br>Ex-smoker: 37 (6.0 %)<br>Non-smoker: 476 (77.5 %)<br>N/A: 7 (1.2 %)	-	-	-	-	-	-
Cancer-free<br>+<br>Medication-free<br>(subgroup 1, n = 237)	53.1	NO: 236<br>(99.6 %)<br>YES: 0<br>(0 %)<br>N/A: 1<br>(< 1 %)	NO: 237<br>(100 %)<br>YES: 0<br>(0 %)<br>N/A: 0<br>(0 %)	NO: 199<br>(84 %)<br>YES: 33<br>(13.9 %)<br>N/A: 5<br>(2.1 %)	N/A: 133<br>165	N/A: 130<br>65.47	Smoker: 33 (13.9 %)<br>Ex-smoker: 13 (5.5 %)<br>Non-smoker: 185 (78 %)<br>N/A: 6 (2.5 %)	-	-	-	-	-	-
Cancer-free<br>+<br>Medication-free<br>(subgroup 2, n = 51)	54.1	NO: 45<br>(88.2 %)<br>YES: 0<br>(0 %)<br>N/A: 6<br>(11.8 %)	NO: 44<br>(86.3 %)<br>YES: 0<br>(0 %)<br>N/A: 7<br>(13.7 %)	NO: 37<br>(72.5 %)<br>YES: 9<br>(17.6 %)<br>N/A: 5<br>(9.8 %)	N/A: 27<br>162	N/A: 25<br>69.63	Smoker: 11 (21.6 %)<br>Ex-smoker: 3 (5.9 %)<br>Non-smoker: 36 (70.6 %)<br>N/A: 1 (1.9 %)	-	-	-	-	-	-
Healthy<br>(independent cohort) (n = 186)	40.5	-	-	-	-	-	-	-	-	-	-	-	-

N/A: Not available data
BC: Breast cancer
*One-way ANOVA test showed that both weight and height are not statistically significant for the discrimination of the any studied groups comparison ($p > 0.2$).

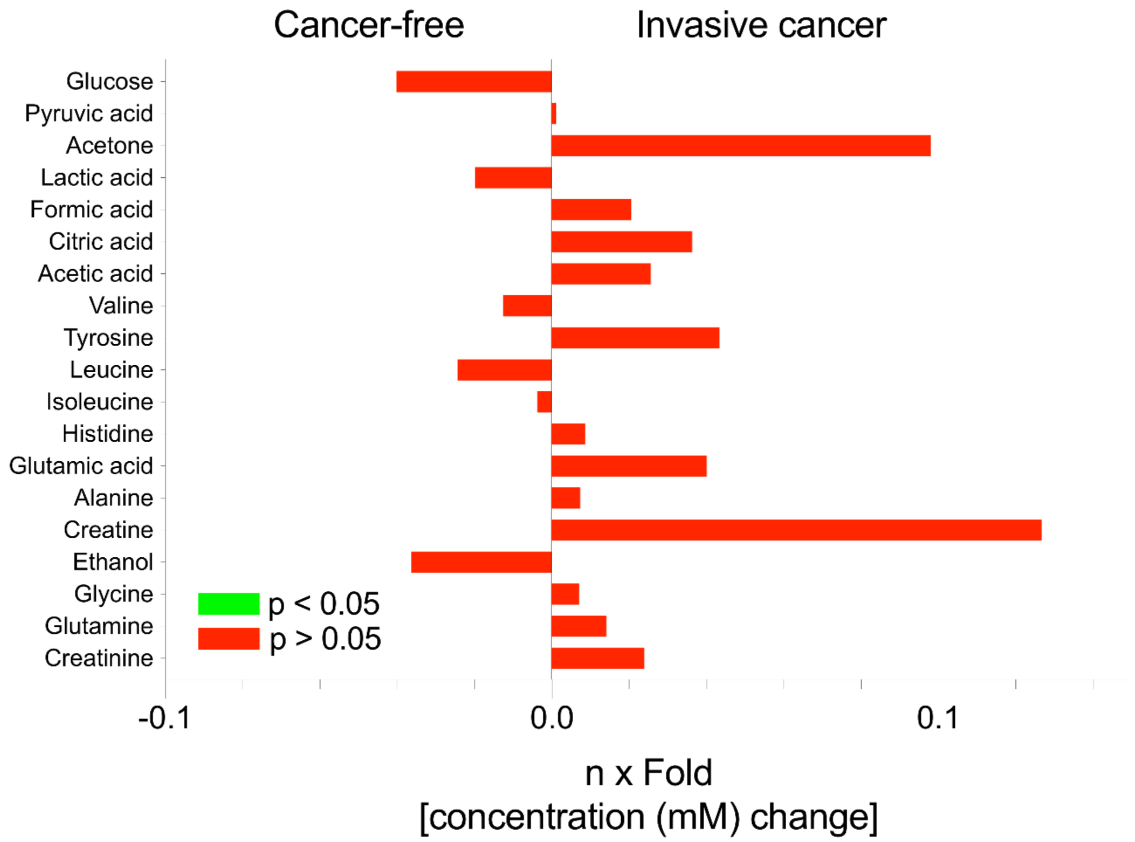

Supplementary Figure 1. The 19 metabolites n x Fold concentration changes between the groups of Cancer-free (n = 614) vs. Invasive cancer. The n x Fold was calculated by the equation: $n \times Fold = log_2\left(\frac{median\ of\ group\ 1}{median\ of\ group\ 2}\right)$. Moreover, one-way ANOVA analysis coupled with t-test was performed for the determination of the statistically significant ($p < 0.05$) differences of the observed metabolites' concentration changes for each case. For each comparison, each metabolite concentration is higher in the group pointed by the bar.

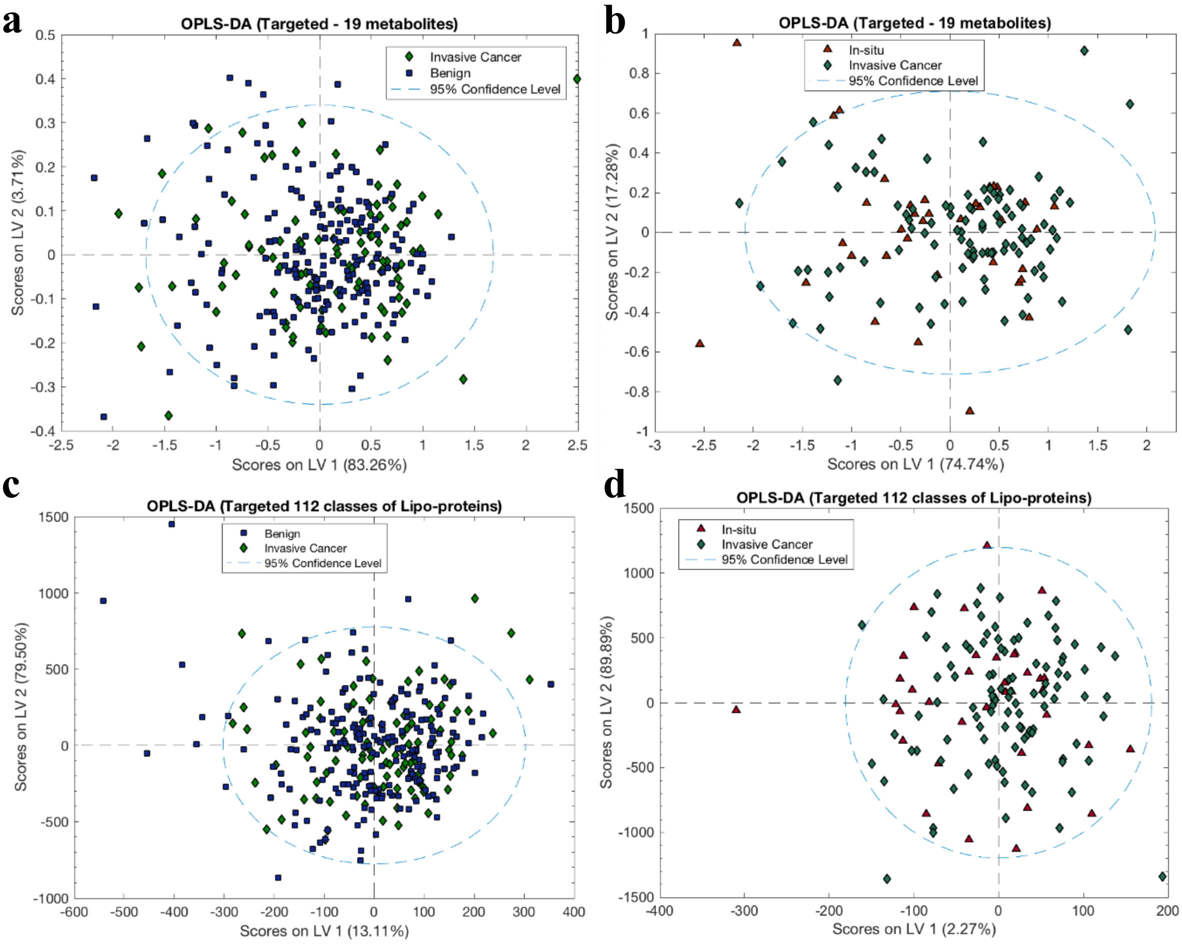

Supplementary Figure 2. (**a-b**) Score-plots of the OPLS-DA models based upon 19 metabolites absolute concentrations for **a.** Invasive cancer vs. benign and **b.** In-situ vs invasive cancer patients. (**c-d**) Score-plots of the OPLS-DA models based upon 112 classes of lipo-proteins absolute concentrations for **c.** Invasive cancer vs. benign and **d.** In-situ vs invasive cancer patients.

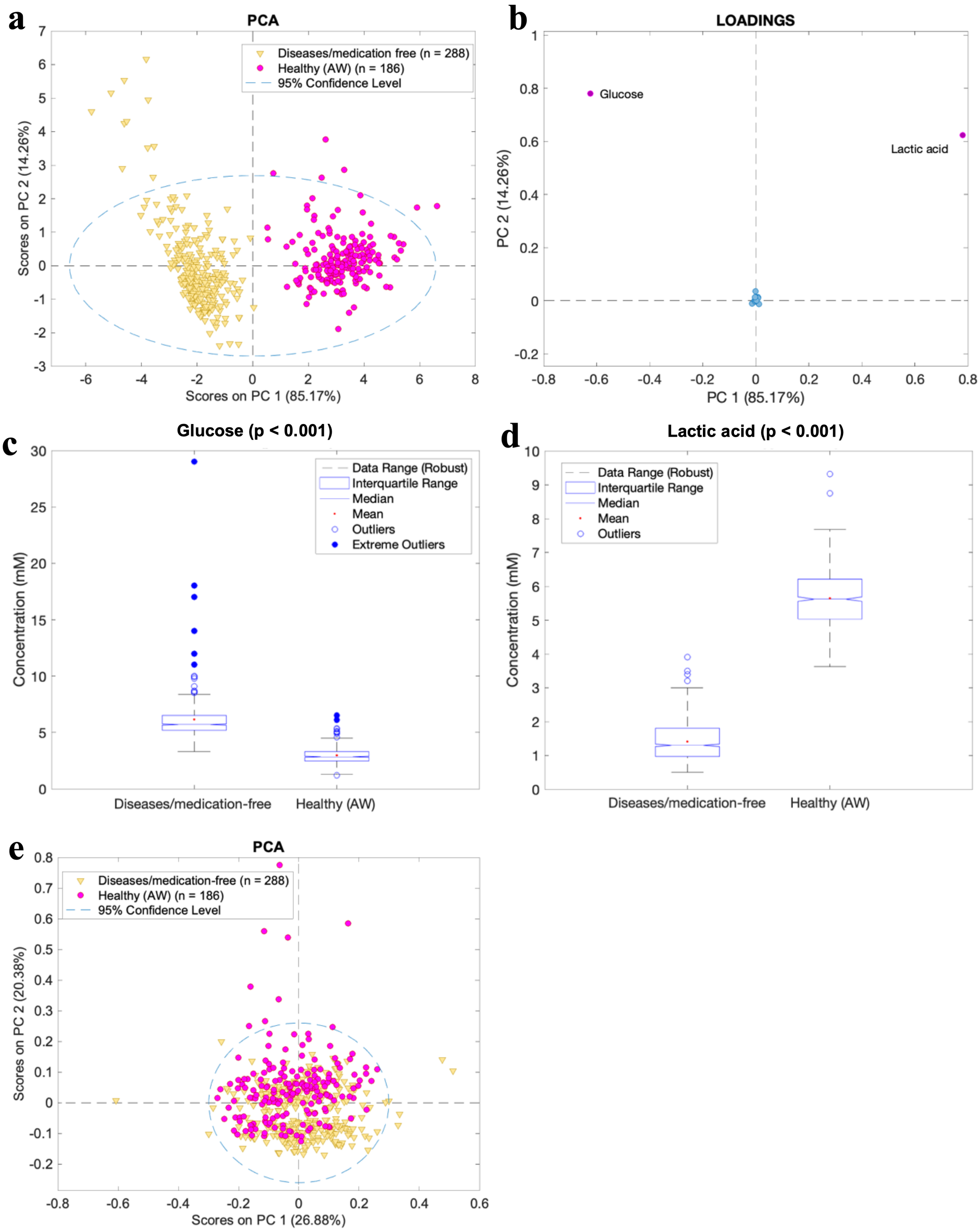

Supplementary Figure 3. **a.** Score-plot based upon 19 metabolites absolute concentrations PCA for the diseases/medication-free of our study and the healthy subjects from the AIRWAVE (AW) project. **b.** PCA loadings plot indicating glucose and lactic acid as the main variables for the two groups discrimination. (**c-d**) One-way ANOVA analysis for glucose and lactic acid concentrations, respectively, indicates that both metabolites are significantly different between the 2 datasets. **e.** Score-plot based upon 17 metabolites (after the removal of glucose and lactic acid) absolute concentrations PCA for the diseases/medication-free of our study and the healthy subjects from the AIRWAVE project

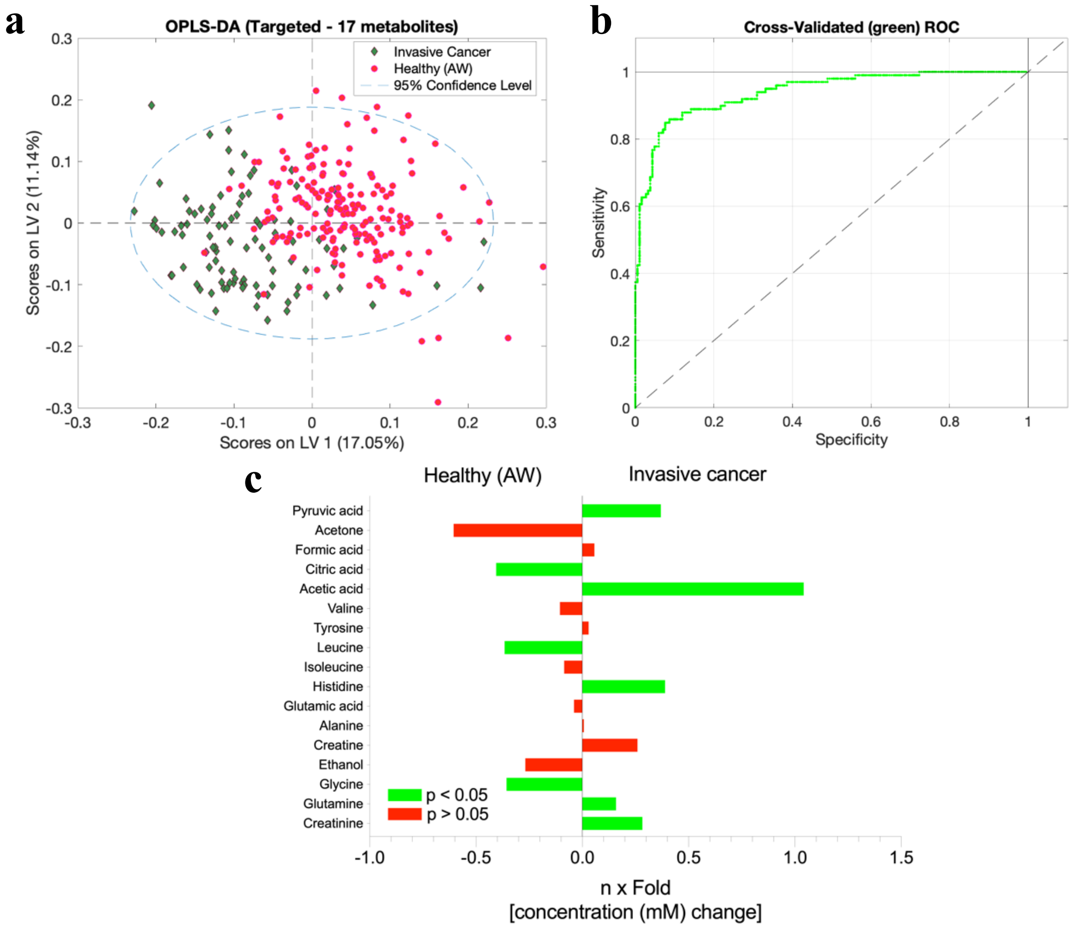

Supplementary Figure 4. a. Score-plot and **b.** Receiver operating characteristic curve produced from 17 metabolites absolute concentrations OPLS-DA analysis for healthy subjects from the AIRWAVE (AW) project versus the invasive breast cancer patients. **c.** The 17 metabolites n x Fold concentration changes between the groups of invasive cancer vs. healthy AW subjects. The n x Fold was calculated by the equation: $n \times Fold = log_2\left(\frac{median\ of\ group\ 1}{median\ of\ group\ 2}\right)$. Moreover, one-way ANOVA analysis coupled with t-test and adjusted for multiple testing using the false discovery rate (FDR) procedure with Benjamini-Hochberg correction was performed for the determination of the statistically significant ($p < 0.05$) differences of the observed metabolites' concentration changes for each case. For each comparison, each metabolite concentration is higher in the group pointed by the bar.

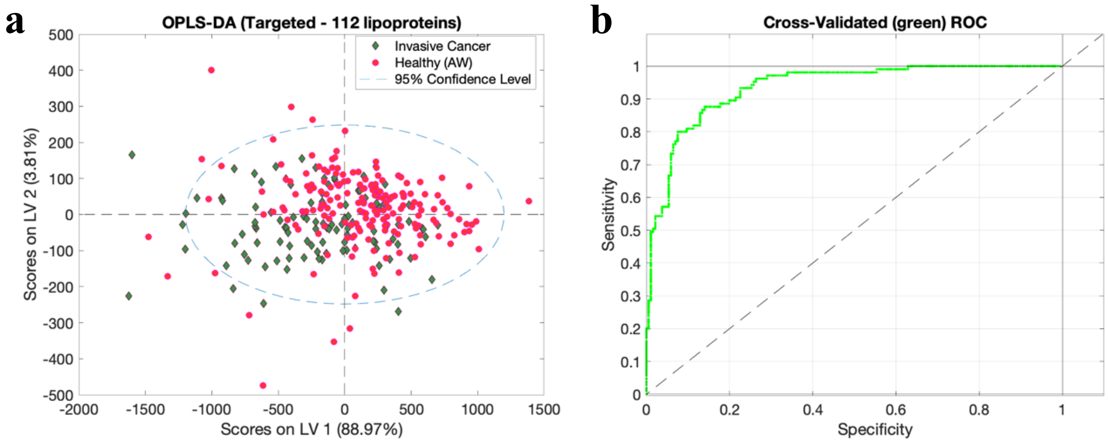

Supplementary Figure 5. a. Score-plot and **b.** Receiver operating characteristic curve produced from 112 plasma lipoproteins absolute concentrations OPLS-DA analysis for healthy subjects from the AIRWAVE (AW) project versus the invasive breast cancer patients.

Supplementary Table 2. The lipoproteins classes which appear as statistically significant ($p < 0.05$) biomarkers for the discrimination of invasive cancer patients vs. the healthy AIRWAVE (AW) subjects.

Lipoproteins[#]	Fold change[+]	
TPCH (Total Plasma Cholesterol)	0.20	
IDCH (Intermediate-density Cholesterol)	0.65	
LDFC (Low-density Free Cholesterol)	0.26	
IDPL (Intermediate-density Phospholipids)	0.63	
V2TG	0.98	Very Low Density Triglycerides
V3TG	0.87	
V4TG	0.47	
V3CH	0.80	Very Low Density Cholesterol
V4CH	0.60	
V5CH	-0.51	
V5FC	-1.50	Very Low Density Free Cholesterol
V2PL	0.84	Very Low Density Phospholipids
V3PL	0.74	
L5FC	0.41	Low Density Free Cholesterol
L6FC	0.59	
H3FC	0.38	High Density Free Cholesterol
H3FC	0.34	

[+]Positive values indicate increased concentration in invasive cancer patients and negative values higher concentration in healthy AW subjects.

[#]Numbering is according to particles increasing density in each lipoprotein subfractions.

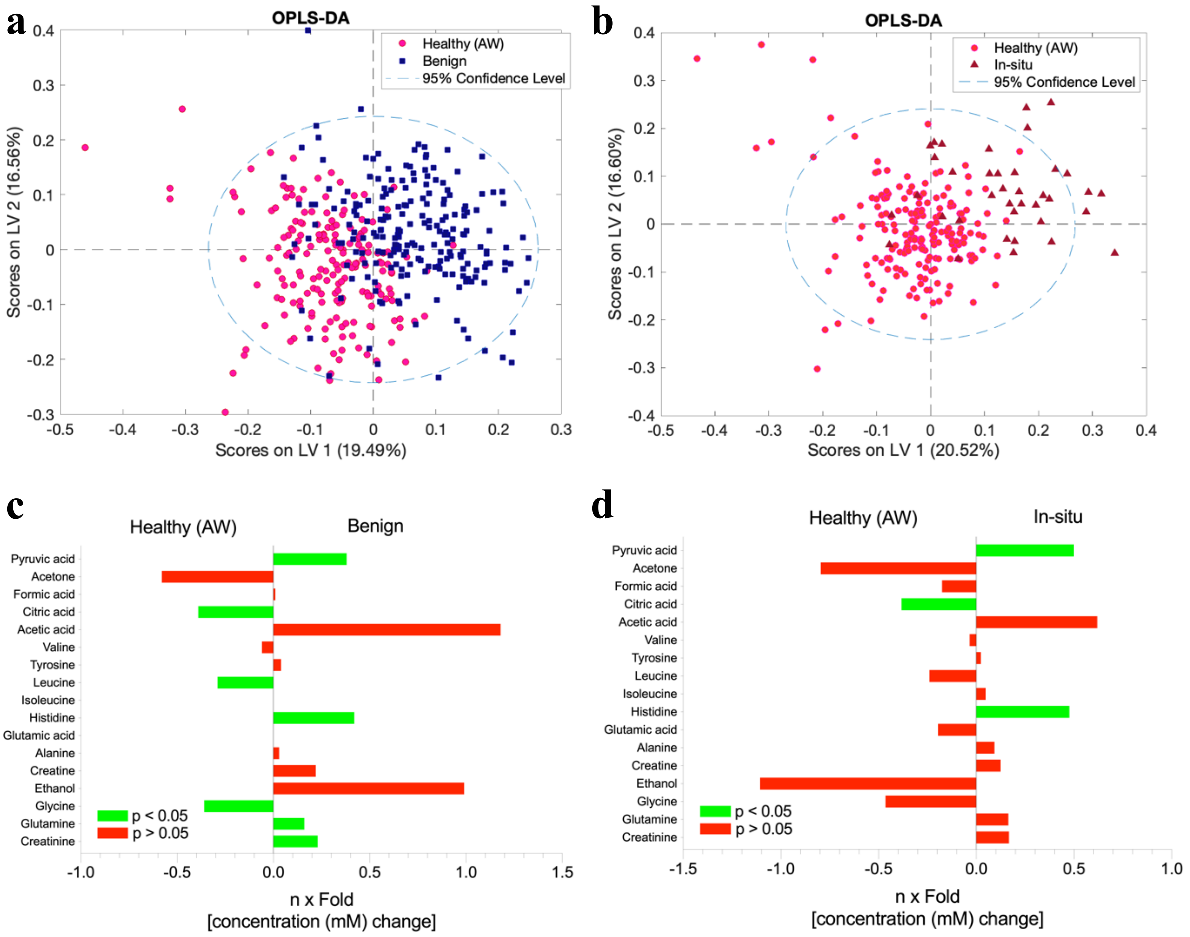

Supplementary Figure 6. **a.** Score-plots of the OPLS-DA models from the **a.** benign vs healthy (AW) and the **b.** In *situ* vs healthy (AW) subjects based upon 17 metabolites concentration values. The 17 metabolites n x Fold concentration changes between the groups of **c.** benign vs. healthy AW subjects and **d.** in-situ vs. healthy AW subjects. The n x Fold was calculated by the equation: $n \times Fold = log_2\left(\frac{median\ of\ group\ 1}{median\ of\ group\ 2}\right)$. Moreover, one-way ANOVA analysis coupled with t-test and adjusted for multiple testing using the false discovery rate (FDR) procedure with Benjamini-Hochberg correction was performed for the determination of the statistically significant ($p < 0.05$) differences of the observed metabolites' concentration changes for each case. For each comparison, each metabolite concentration is higher in the group pointed by the bar.

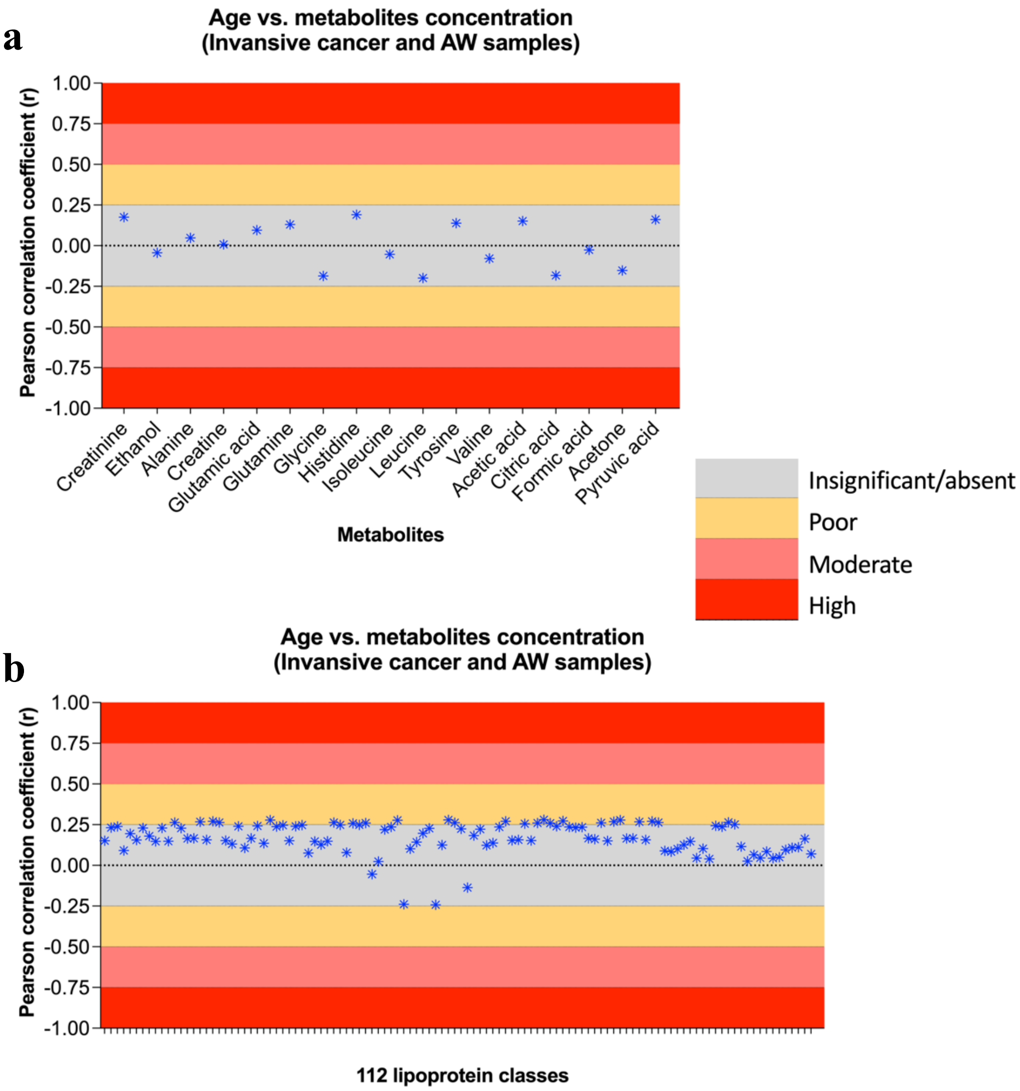

Supplementary Figure 7. The calculated Pearson coefficients from the correlation of BSMS invasive cancer (n = 105) and AW (n = 186) subjects' age and the (**a**) 17 plasma metabolites concentrations, (**b**) 112 lipoprotein classes, all measured by NMR. As depicted, there is insignificant (or absent) correlation between subjects' age and these parameters in our datasets.

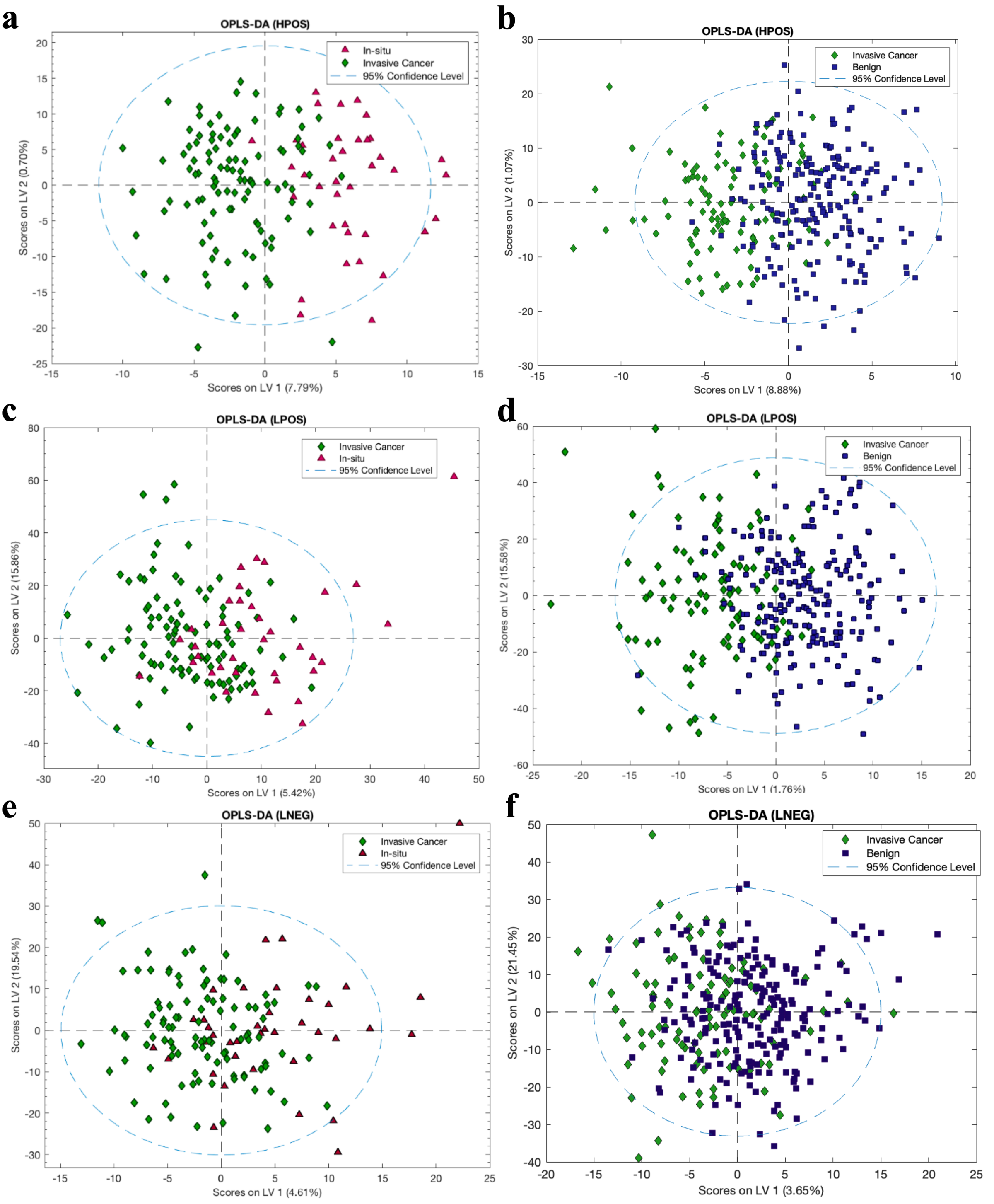

Supplementary Figure 8. (**a-b**) Score-plots of the OPLS-DA models based upon HILIC+ (HPOS)MS assays for **a.** In-situ vs invasive cancer and **b.** Invasive cancer vs. benign tumour patients. (**c-d**) Score-plots of the OPLS-DA models based upon Lipid RPC+ (LPOS) MS assays for **c.** In-situ vs invasive cancer and **d.** Invasive cancer vs. benign tumour patients. (**e-f**) Score-plots of the OPLS-DA models based upon Lipid RPC- (LPOS) MS assays for **e.** In-situ vs invasive cancer and **f.** Invasive cancer vs. benign tumour patients.

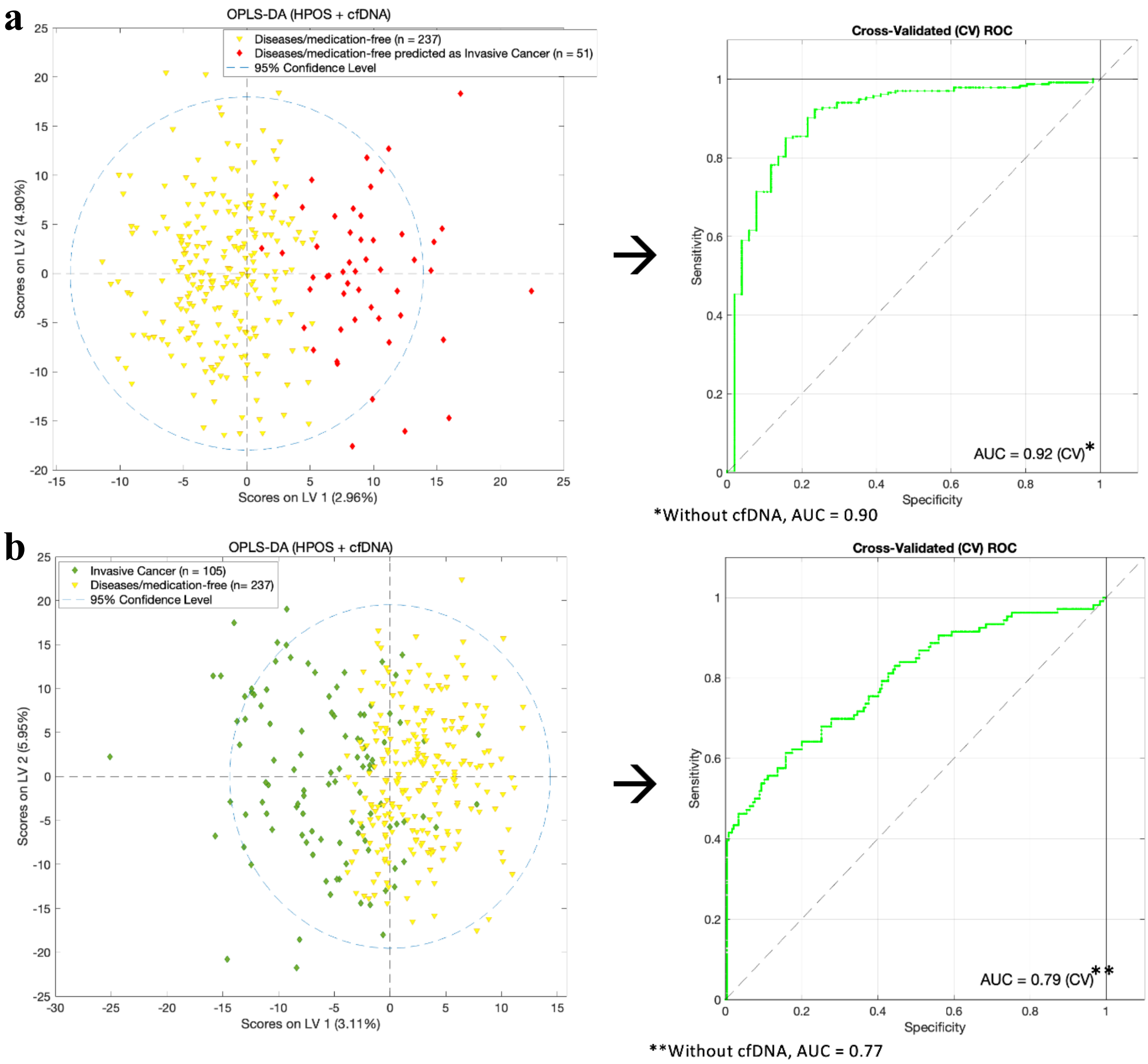

Supplementary Figure 9. Score-plots of the OPLS-DA models along with the cross-validated ROC curves based upon HILIC+ (HPOS) MS assay combined with cfDNA measurements for: (**a**) Disease/medication-free (subgroup 1, n = 237) vs Disease/medication-free (subgroup 2, n = 51). The AUC value (0.92) is slightly increased compared to the model from only the HILIC+ (HPOS) MS assay dataset (0.90, see Table 1). (**b**) invasive cancer (n = 105) vs disease/medication-free (subgroup 1, n = 237). The AUC value (0.79) is slightly increased compared to the model from only the HILIC+ (HPOS) MS assay dataset (0.77, see Table 1). Overall, the cfDNA data marginally improve the predictability of metabolomics data only based models.

Supplementary references

1. Lewis MR, Pearce JTM, Spagou K, et al: Development and Application of Ultra-Performance Liquid Chromatography-TOF MS for Precision Large Scale Urinary Metabolic Phenotyping. Anal Chem 88:9004–9013, 2016

2. Izzi-Engbeaya C, Comninos AN, Clarke SA, et al: The effects of kisspeptin on β-cell function, serum metabolites and appetite in humans. Diabetes, Obes Metab 20:2800–2810, 2018

3. Smith CA, Want EJ, O'Maille G, et al: XCMS: Processing Mass Spectrometry Data for Metabolite Profiling Using Nonlinear Peak Alignment, Matching, and Identification. Anal Chem 78:779–787, 2006

4. Sands CJ, Wolfer AM, Correia GDS, et al: The nPYc-Toolbox, a Python module for the pre-processing, quality-control and analysis of metabolic profiling datasets. Bioinformatics , 2019

5. Dona AC, Jiménez B, Schäfer H, et al: Precision High-Throughput Proton NMR Spectroscopy of Human Urine, Serum, and Plasma for Large-Scale Metabolic Phenotyping. Anal Chem 86:9887–9894, 2014

6. Jiménez B, Holmes E, Heude C, et al: Quantitative Lipoprotein Subclass and Low Molecular Weight Metabolite Analysis in Human Serum and Plasma by 1H NMR Spectroscopy in a Multilaboratory Trial. Anal Chem 90:11962–11971, 2018

7. Page K, Guttery DS, Zahra N, et al: Influence of Plasma Processing on Recovery and Analysis of Circulating Nucleic Acids. PLoS One 8:e77963, 2013
